# The Duties of Hospital Physicians and Surgeons

**Published:** 1868-07

**Authors:** 


THE DUTIES OF HOSPITAL PHYSICIANS AND SURGEONS.—The needless increase of free dispensary and hospital treatment is an abuse. It is a vital injury to the young physician, who must live on small fees obtainable from just those middling classes of the community whom the dispensary system invites to a gratuitous treatment.

We hold it, therefore, to be strictly the duty of the dispensary physician, or the physician to out-patients at a hospital, to distinguish carefully between those applicants who should pay something and those who cannot; and either to exclude the former, or to enforce the payment of suitable fees for the support of the charity which is to feed the poorest class.

We hold, also, that it is the right and duty of the hospital staff to be paid for their services, a moderate salary; not in proportion to the work they do, for no hospital could afford that; but just enough to furnish an acknowledgment of the fact that their services are recognized and compensated.

Such a claim could not be considered venal or narrow on the part of our profession, unequaled and unapproached by any calling in life in the amount of gratuitous service it unavoidably and cheerfully renders to the world.—*Boston Medical and Surgical Journal.*

